# NIA DIVISION OF BEHAVIORAL AND SOCIAL RESEARCH

**DOI:** 10.1093/geroni/igad104.0834

**Published:** 2023-12-21

**Authors:** Lisbeth Nielsen

**Affiliations:** National Institute on Aging, Bethesda, Maryland, United States

## Abstract

Dr. Nielsen will discuss research priorities for the NIA Division of Behavioral and Social Research. Dr. Nielsen will also be available for small group discussion.

